# Ultrasonic Cutting and Coagulating Device in Implant-based Breast Reconstruction

**DOI:** 10.1097/GOX.0000000000002020

**Published:** 2018-11-01

**Authors:** Alessio Baccarani, Marta Starnoni, Giorgio De Santis

**Affiliations:** From the Department of Medical and Surgical Sciences, Division of Plastic Surgery, University of Modena and Reggio Emilia, Modena, Italy.

Dear Sir,

We have found a lot of interest in using ultrasonic cutting and coagulating instrument (Harmonic Scalpel, Johnson and Johnson, New Jersey). This multifunctional device presents a few advantages when compared with traditional electrocoagulation including tissue dissection and hemostasis at the same time, no eschar formation over the blade, minimal thermal damage, no smoke formation, no risk of electrical injury, and possibility of use in patients with pacemaker. Its use has been successfully applied in minimally invasive laparoscopic procedures and in head and neck surgery.

We have focused our attention on the use of Harmonic Scalpel in breast surgery. With respect to mastectomy, the literature is varied, with some studies showing less serous drainage and blood loss^1^ and others showing no differences.^2^ With respect to breast reconstruction, its use has been reported in latissimus dorsi and in deep inferior epigastric perforator flap harvest.^3,4^ We have experienced this surgical instrument for the dissection of the submuscular pocket in immediate implant-based breast reconstruction (both with tissue-expander and direct-to-implant) observing significant differences between the use of Harmonic Scalpel and traditional electrocoagulation.

The surgical technique for the creation of the subpectoral pocket is the same with the use of Harmonic Scalpel or electrocautery. The pectoralis major muscle is dissected starting from its lateral border towards the medial sternal insertion, exposing inferiorly the aponeurosis of the anterior rectus and superiorly arriving up to the second rib. The serratus anterior muscle is also dissected to complete the lateral part of the submuscular pocket. Two drainages are placed respectively in the submuscular and subcutaneous plane.

We compared 2 groups of 20 patients: group A (creation of the pocket with Harmonic Scalpel) and group B (creation of the pocket with electrocautery). The most important differences between the 2 groups were about the total drainage volume (125.50 mL in group A and 165.50 in group B), the drainage removal (in second postoperative day in group A and in third postoperative day in group B) and the pocket creation operative time (22 minutes in group A and 38 minutes in group B).

The difference in operative time is possibly explained by the absence of hemostasis time with the use of the Harmonic Scalpel and the lower fluid formation may be related to the extreme accuracy of vessel coagulation.

The use of the Harmonic device for the dissection of the pectoralis major muscle flap was described by Deo et al.^5^ for head and neck reconstruction and, according to our results, shorter dissection time and lower total drainage volume are reported.

Despite limited evidences, our good results will allow us to continue with the use of this surgical device in the future.

Furthermore, and less importantly, the use of Harmonic Scalpel improves self-confidence in our junior residents during the medial dissection of the pocket, where care should be taken not to injure the thoracoacromial vessels or the perforators of the internal mammary artery where a sudden bleeding may be of difficult management.
